# Processing of pragmatic communication in ASD: a video-based brain imaging study

**DOI:** 10.1038/s41598-020-78874-2

**Published:** 2020-12-10

**Authors:** Aija Kotila, Aapo Hyvärinen, Leena Mäkinen, Eeva Leinonen, Tuula Hurtig, Hanna Ebeling, Vesa Korhonen, Vesa J. Kiviniemi, Soile Loukusa

**Affiliations:** 1 Research Unit of Logopedics, Faculty of Humanities, University of Oulu, Oulu, Finland; 2 Department of Computer Science, University of Helsinki, Helsinki, Finland; 3 Office of the Vice Chancellor, Murdoch University, Murdoch, WA Australia; 4 Research Unit of Clinical Neuroscience, Psychiatry, University of Oulu, Oulu, Finland; 5 PEDEGO Research Unit, The Faculty of Medicine, University of Oulu, Oulu, Finland; 6 Department of Child Psychiatry, Faculty of Medicine, Institute of Clinical Medicine, Oulu University Hospital, Oulu, Finland; 7 Department of Diagnostic Radiology, Medical Research Center (MRC), University and University Hospital of Oulu, Oulu, Finland; 8 Research Unit of Medical Imaging, Physics and Technology, The Faculty of Medicine, University of Oulu, Oulu, Finland

**Keywords:** Neurology, Signs and symptoms, Neuroscience, Cognitive neuroscience, Social behaviour, Social neuroscience

## Abstract

Social and pragmatic difficulties in autism spectrum disorder (ASD) are widely recognized, although their underlying neural level processing is not well understood. The aim of this study was to examine the activity of the brain network components linked to social and pragmatic understanding in order to reveal whether complex socio-pragmatic events evoke differences in brain activity between the ASD and control groups. Nineteen young adults (mean age 23.6 years) with ASD and 19 controls (mean age 22.7 years) were recruited for the study. The stimulus data consisted of video clips showing complex social events that demanded processing of pragmatic communication. In the analysis, the functional magnetic resonance imaging signal responses of the selected brain network components linked to social and pragmatic information processing were compared. Although the processing of the young adults with ASD was similar to that of the control group during the majority of the social scenes, differences between the groups were found in the activity of the social brain network components when the participants were observing situations with concurrent verbal and non-verbal communication events. The results suggest that the ASD group had challenges in processing concurrent multimodal cues in complex pragmatic communication situations.

## Introduction

Communication in everyday interaction requires flexible processing of constantly changing social and linguistic signals and related contextual information. In addition to verbal utterances in face-to-face communicative situations, facial expressions and body language are inseparable components of communication and, therefore, interpretation of these non-verbal cues is essential when drawing pragmatic inferences^1^. In real-life situations, information processing is challenging because of the necessity to process multimodal information often from multiple people at the same time. In addition to cues requiring perception capabilities, the interpretation of complex contextual and mental cues, such as intentions of the interlocutors, needs to be taken into account when inferencing the pragmatic meaning in a communicative situation^2^.

Autism spectrum disorder (ASD) is characterized by difficulties in social communicative function, repetitive behaviours, and restricted interests^3^. As pragmatic communication is one of the core factors of social communication, it is obvious that pragmatic communication deficits are one component of the features of ASD^4^. Hitherto, most of the pragmatic communication studies in ASD have been carried out with children and adolescents^5^. However, there are some studies with adults showing that difficulties in pragmatic communication processing may persist into adulthood in individuals with ASD^6,7^. In addition, there are not enough studies focusing on multi-level contextual processing in ASD, meaning understanding the demands of attention directing, monitoring and connecting various pieces of information at the same time, although this kind of processing is often needed in real-life communication situations.

Some evidence exists that pragmatic impairment in ASD is related to diminished capability of comprehending rapidly changing socially salient cues, which suggests processing difficulties^8^. It has also been argued that an inability to take the communication context into account in ASD stems from poor cognitive flexibility^9^. In addition to domain general processing difficulties, symptoms of ASD have been linked to impaired perception of cues such as different valences from faces^10^ or communicative gestures^11^. Furthermore, low pragmatic inference abilities in ASD may be linked to predicting and inferring consequences from others’ movements^12^, which may be related to deficits in taking an allocentric stance when observing others^13^.

Earlier, it was suggested that pragmatic communication is mostly mediated by the right brain hemisphere^14^. Nowadays, based on functional magnetic resonance imaging (fMRI) studies, it is thought that there are certain large-scale brain networks that are associated with social and pragmatic information processing^15–17^. One of these networks is the salience network covering areas of the insula and the anterior cingulate cortex and mediating salient event detection^18^. It is believed that regions of the salience network have a role in attributing mental states to others^16^, which is connected to pragmatic language comprehension^19^. Moreover, the salience network is thought to support agile task processing by mediating switching between cognitive resources^20^. In addition to the salience network, another network that may also affect pragmatic information processing is the default mode network (DMN)^21^ that has been associated with making social inferences^15^ and understanding of others^22^.

The symptoms of ASD have been linked to atypical connectivity and neural activation of the salience network^23,24^ and the DMN^25^. When looking at the brain regions belonging to the salience network, the link between a dysfunctional anterior insula and abnormal activity of the anterior cingulate cortex in ASD has also emerged in several studies^26,27^. Furthermore, the anterior cingulate cortex has a role in social decision making, responding to other-oriented information and tracking the motivation of others^28^.

Although there is increasing knowledge about neural activity linked to various fragments of social and pragmatic communication processing, little is known about brain function when observing pragmatically challenging communication situations. Compared to pictures or short sentences, naturalistic movie stimuli can better reveal the underlying causes of well-known difficulties that individuals with ASD are facing in everyday pragmatic communication situations. Using movies as a stimulus also reduces head motion and improves arousal during fMRI scanning^29^. Moreover, altered brain network functionality possibly linked to multisensory integration problems has been observed in subjects with ASD while they have been watching specific movie sub-parts^30^. However, movie stimulus has not been widely used in ASD studies, and there is an obvious lack of understanding concerning pragmatic communication processing in ASD. The results of previous studies relating to typically functioning individuals suggest that hemodynamic changes and brain activity become synchronized between viewers during movie viewing^31–33^, and that the test/retest reliability of connectivity measures of brain networks is higher during naturalistic movie viewing than in resting state conditions^34^. Since the variability of network components between individuals is low in visual and somatomotor networks during movie viewing^32^, this research focuses on social brain network components, which we suggest better reveal differences in pragmatic processing between ASD and neurotypical (NT) groups.

This study utilizes fMRI and movie stimulus to capture the neural level processing of young adults with ASD when viewing naturalistic and pragmatically complex social situations played by actors. Since social communication difficulties are a part of ASD^3^, we hypothesize that these difficulties should also be visible in the brain level processing. Therefore, the first aim of the study is to explore the response of the social brain network components of the participants to the complex social episodes in order to compare young adults with ASD and NT controls. If differences between the groups are found in the brain network component activity, the second aim of the study is to analyse the pragmatic communication and social interaction aspects of the events that evoke the statistically significant differences between the groups when comparing the responses of the selected social network components.

## Results

### Timecourses of the selected independent brain network components

**Figure 1:**
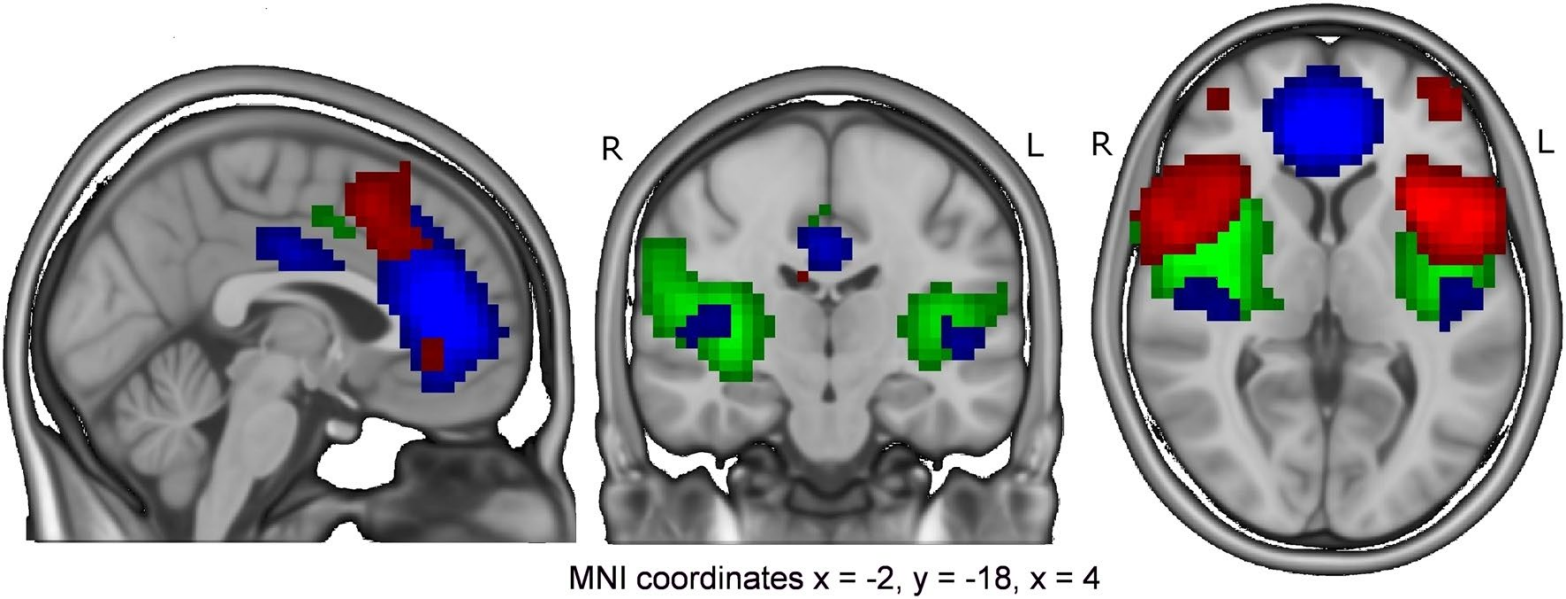
Regions of the selected independent components (green = IC4, blue = IC15, red = IC27).

**Figure 2:**
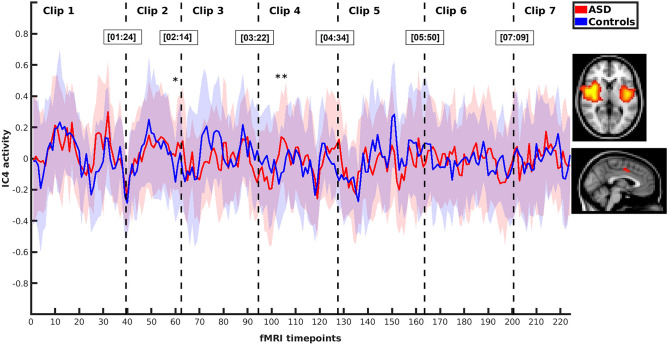
Average activity (± *SD*) of IC4 in the ASD and NT groups during video clips 1–7 (* = significant difference after combining probability over IC4, IC15 and IC27).

**Figure 3:**
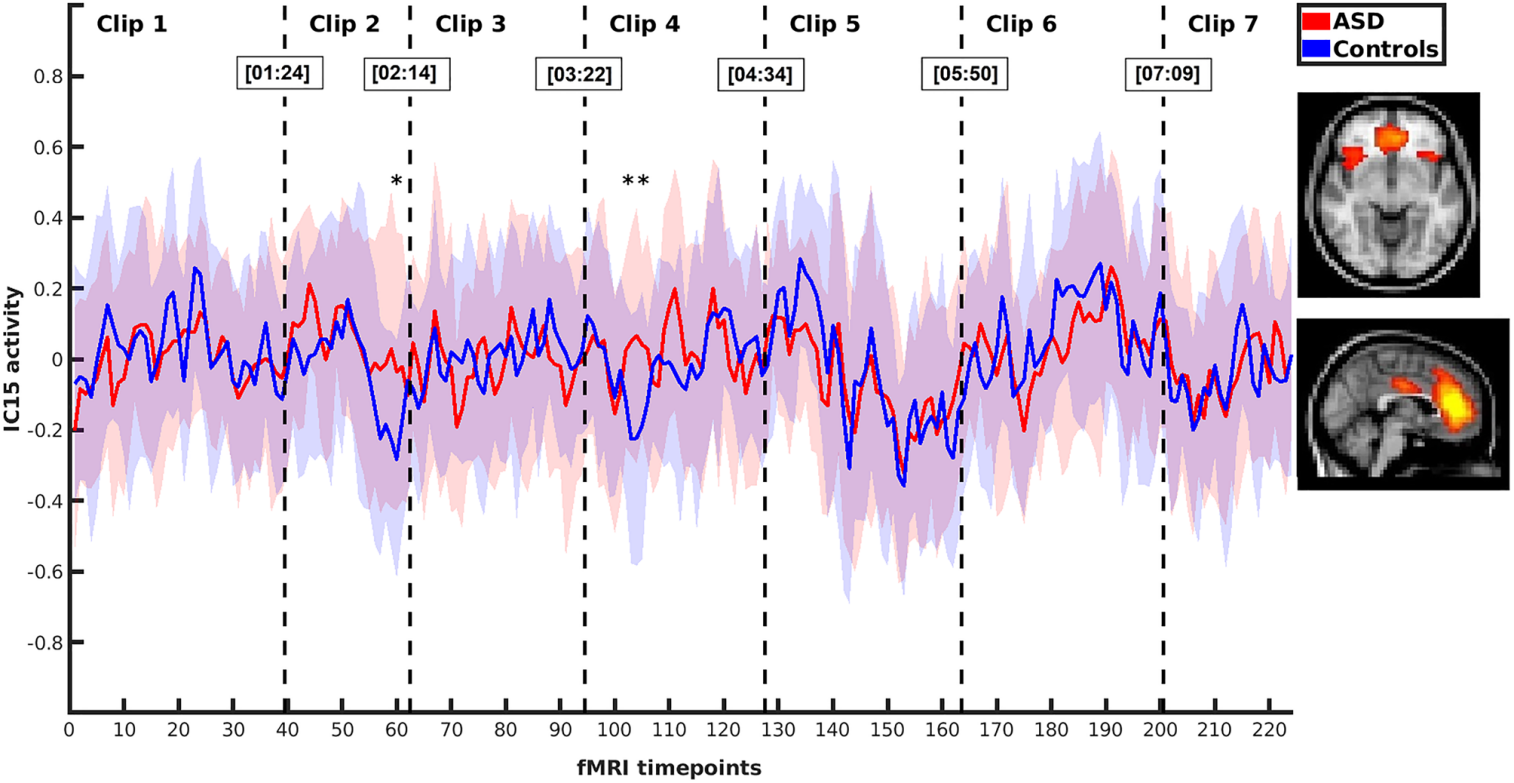
Average activity (± *SD*) of IC15 in the ASD and NT groups during video clips 1–7 (* = significant difference after combining probability over IC4, IC15 and IC27).

**Figure 4:**
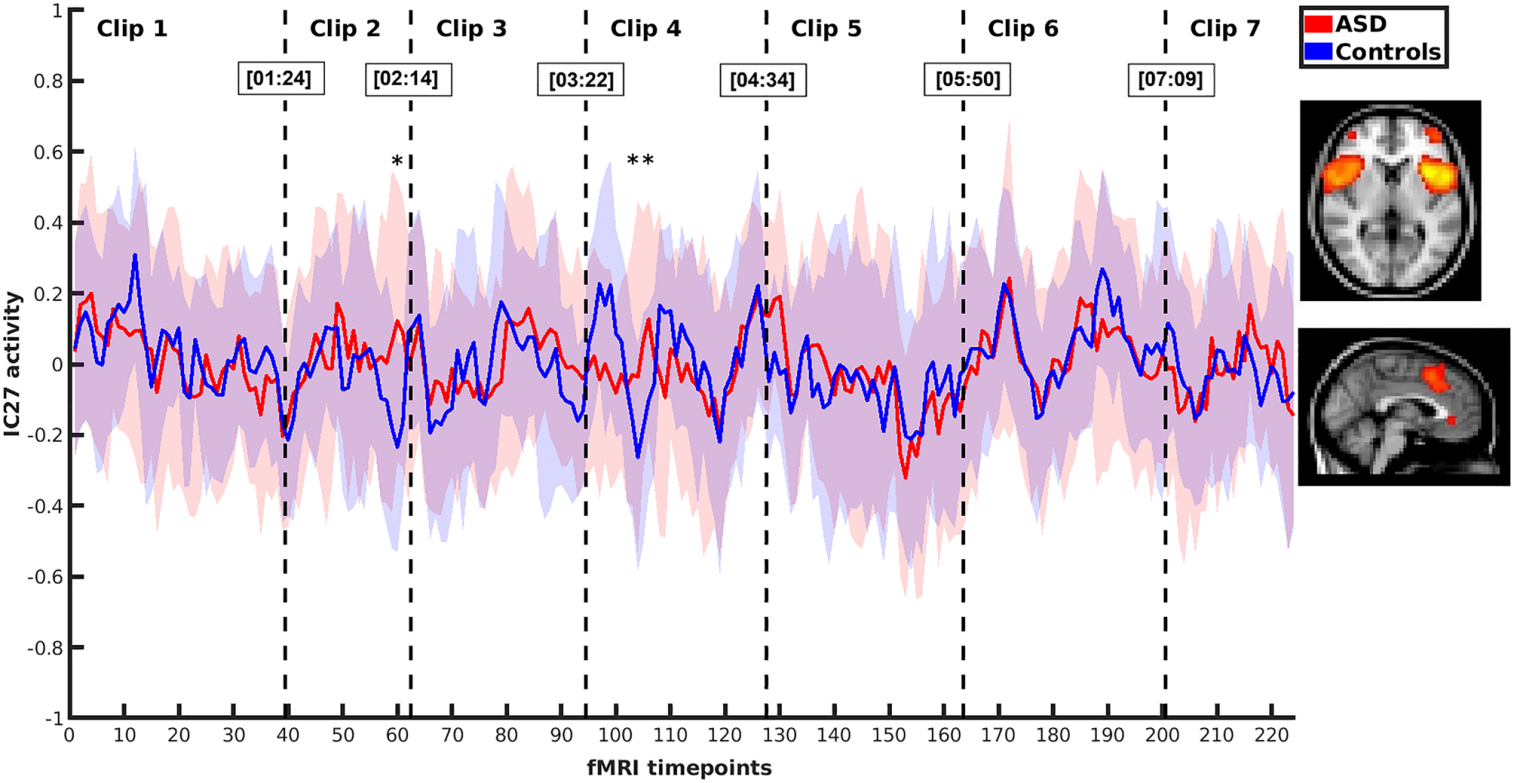
Average activity (± *SD*) of IC27 in the ASD and NT groups during video clips 1–7 (* = significant difference after combining probability over IC4, IC15 and IC27).

Altogether 70 time-independent brain network components were identified using independent component analysis. For this study, three independent components, IC4, IC15 and IC27, were chosen manually based on their spatial patterns (Table 1 and Fig. 1). These components were selected for their assumed role in pragmatic and social understanding^17^. Two of the selected components, IC4 and IC27, were located mostly in the insular cortex representing the salience network. The third independent component, IC15, included regions of the salience network, e.g. the anterior cingulate cortex and the insular cortex, also spreading to areas typical for the anterior DMN^21^. In order to reveal the differences in brain response between the ASD and NT groups while viewing pragmatically and socially challenging stimulus data in seven video clips, comparison of timecourses of selected independent brain network components was performed between the ASD and NT groups. Average timecourses of these independent components for the ASD and NT groups can be found in Figs. 2, 3 and 4. The figures show that the average timecourses were predominantly similar between the groups for all 3 components. However, there were local differences in certain timepoints of the fMRI data when considering all three independent components together.

**Table 1:** Description of the selected independent components (ICs).

Component	Main brain regions
IC4	L + R insular cortex, L opercular cortex, anterior cingulate cortex
IC15	anterior cingulate cortex, R insular cortex, L + R inferior frontal gyrus, L + R primary auditory cortex
IC27	L + R insular cortex, paracingular cortex

R, right; L, left.

### Analysis of the timecourses in order to select video clips for further inspection

The next step of the study was to analyse whether there were statistically significant differences between the groups when comparing the timecourses of the independent components IC4, IC15 and IC27 together. A statistically significant difference between the ASD and NT groups was found in timepoint 60 (*χ*^2^(6) = 24.10, *p* < 0.001) in the video clip 2, and also in two consecutive timepoints 104 (*χ*^*2*^(6) = 20.34, *p* = 0.002) and 105 (*χ*^2^(6) = 20.50, *p* = 0.002) in the video clip 4 after calculation of Fisher combined probability over IC4, IC15 and IC27 network components and FDR for multiple comparison correction over the fMRI timepoints of the clips 2 and 4, respectively. The timepoints of significant difference are marked with * in Figs. 2, 3 and 4. There was no statistically significant difference between the groups for any timepoints in the video clips 1, 3, 5, 6 or 7 after combined probability over IC4, IC15 and IC27 and FDR correction.

### Group differences in independent component activity linked to pragmatic content

Video clips 2 and 4 were further analysed qualitatively in order to examine pragmatic communication content and social interaction aspects linked to those timepoints where statistically significant differences between the groups were found. All of the seven video clips included complex social situations, but there was one common denominator between the communication events in the clips 2 and 4 where the differences were found. In both of these clips there was an episode of two overlapping communication events, one with verbal and the other with non-verbal communication, requiring attention shifting between the events. The other video clips 1, 3, 5, 6 or 7 did not include parallel communication events, but had one-to-one or one-to-many verbal communication concerning a single topic at a time.

In the video clip 2 two women, namely the grandmother (*Senni*) and her daughter (*Marja*) are having a power struggle over organization of the kitchen, while *Marja*’s daughter (*Roosa-Maria*) enters the room with her dog. The two women carry on fighting without noticing *Roosa-Maria*, who quietly gestures to the dog as if to say they should leave the room together in order not to get involved in the fight. Regarding the significant difference between the ASD and NT groups found in the fMRI timepoint 60, response of the independent brain components, when taking into account the response latency of the BOLD signal, is related to the following events in clip 2 (see Supplementary Data D1 for full transcription):

**(1)**
[01:57]: **Marja (mother)**: THERE IS NO SENSE IN THIS AT ALL ((*Marja’s voice can be heard in the background while the camera shows Roosa-Maria, who makes a silence gesture to the dog with one finger on her lips*)).
[01:59]: **Senni (grandmother)**: well I thought that when you are cooking- ((*Senni’s voice can be heard but the camera shows Roosa-Maria, who whispers something to the dog*)).
[02:00]: **Marja**: = DON’T THINK (.) this is MY order here ((*Marja’s voice can be heard in the background while the camera shows Roosa-Maria, who pulls the dog by the collar to leave the room quietly*)).
[02:02]: **Marja**: = because this is MY kitchen (.) have you forgotten that? ((*the camera now shows Marja and Senni facing each other, Roosa-Maria is no longer visible*)).

In video clip 4 the son (*Illi*) of the family enters his home with his new girlfriend (*Ilona*) and the couple wishes to have a private moment. However, the little sister (*Roosa-Maria*) distracts the couple with her stories about a horse (*Sulo*). The couple agrees to go upstairs together using silent gestures for communication while *Roosa-Maria* continues telling her stories in front of the mirror. Regarding the significant difference between the ASD and NT groups found in timepoints 104–105, response of the independent brain components is related to the following events in the clip 4 (see Supplementary Data D2 for full transcription):

**(2)**
[03:39–03:45]: **Roosa-Maria (daughter)**: no (0.7) you know something Illi? (0.9) Sulo has had horse colic several times ((*Roosa-Maria speaks in front of the mirror while she is adjusting her riding helmet; meanwhile Illi nods meaningfully to Ilona and takes her by the hand. Ilona glances at Roosa-Maria to make sure she does not notice what is going on between the couple. The couple sneaks secretly upstairs behind Roosa-Maria's back.*)).

**Figure 5:**
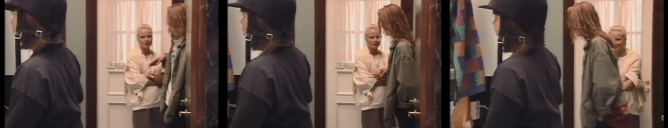
Non-verbal gesture- and gaze-based communication during the events (2) in the video clip 4 (picture published with the permission of Finnish commercial media operator (MTV)).

The descriptions (1) and (2) of the events in video clips 2 and 4 show that there were two overlapping communication events, with simultaneous verbal communication related to one topic and non-verbal gesture- and gaze-based communication related to another topic, occurring just before the timepoints of statistical difference in independent component activity between the groups. Observation of the scenes required a shifting of perspective between two communication events, in which one was carried out verbally and the other non-verbally. The two overlapping communication events in the video clip 4 can be seen in Fig. 5.

## Discussion

The present study explored temporal responses of three brain network components related to social and pragmatic comprehension in order to examine processing of complex communication situations in ASD. The timecourses of the selected brain network components had for the most part similar trends throughout the seven video clips in the ASD and NT groups, showing perhaps predominantly similar processing of social events. This may arise due to the participants’ profile being in the mildest end of the spectrum in the ASD group. However, there were statistically significant differences between the groups in certain timepoints of the timecourses in two video clips. Differences in brain responses found in these timepoints seemed to be related, in a qualitative analysis, to the observation of two overlapping communication events requiring simultaneous comprehension of verbal communication related to one topic and non-verbal communication related to another topic. Similar parallel communication events were not present in the other five video clips, which all instead involved communication about a single topic at a time in either one-to-one or one-to-many settings.

Differences between the ASD and NT groups in social understanding areas of the brain may be a sign of deviant processing in the ASD group when observing pragmatically challenging communication situations^35^. It has been demonstrated earlier that individuals with ASD have atypical brain synchronization when viewing social interaction^36^. In addition, there is evidence that cues with high communicative value require more mental effort from individuals with ASD^11^, which can perhaps explain the difference in neural response between the groups for events requiring interpretation of mental states together with concurrent verbal and non-verbal communication. Furthermore, impairment in social attention is higher when social or pragmatic content is higher, for example when multiple persons are involved in a communicative situation^37^. It should be noted that people with fluent skills typically use less mental effort than less skilled persons^38^.

Group differences in the brain network component responses may be related to atypical functionality of the salience network in ASD^23,24^. The salience network which comprises brain regions such as the insula and the dorsal anterior cingulate cortex has been linked to attributing salience to the perceived external events^18^, and may consequently have a role in pragmatic understanding. For example, abnormal temporal structure of the salience network activity has been observed in resting state conditions in adults with high-functioning ASD^26^, in which the relative predominance of lower over higher frequencies suggests low adaptation to changes and thus, difficulty in switching salience attribution from one communication event or modality to another. Moreover, some papers have linked atypical functionality or connectivity of the anterior insula to ASD^39,40^. As the insula has been associated with the social emotive component in gesture comprehension^41^, differences in neural activation of the insula area are possibly linked to differences in understanding the intentions of others through their actions^12^. The other area of the salience network, namely the anterior cingulate cortex, has been associated with tracking the motivation of others^28^, which is an essential ability when making pragmatic inferences. In addition, more activation has been found in the anterior cingulate cortex in theory of mind tasks in ASD versus typically developing children, possibly indicating increased effort among individuals with ASD^16^.

Interestingly, this study found that the timepoints of statistical difference were related to video clips, in which differences between the groups were associated with sections which included observation of other-oriented non-verbal communication, and concurrent verbal dialogue (clip 2) or monologue (clip 4). Although the processing of the young adults with ASD was similar to that of the NT group during the majority of the video scenes, individuals with ASD may have experienced extra processing load during these events that required perspective shifting and making inferences of mental states from two parallel events. Brain level findings of this study are in line with previous behavioural level findings, suggesting that individuals with ASD have challenges in perspective shifting^13^, which may be related to cognitive inflexibility^9^. Furthermore, it has been reported that there are deficits in cross-modal processing including overlapping spoken information and iconic gesturing in adolescents with high-functioning ASD^42^, which also supports our findings. Because we found atypical processing in individuals with ASD linked to observing parallel communication events including multiple simultaneous contextual elements, we suggest that this may have a connection to difficulties found in ASD concerning pragmatic inference abilities^5^ including reasoning about the mental state of others^19,43^. Our result also implies that pragmatic comprehension skills of individuals with ASD are particularly vulnerable in communication situations involving multiple persons and overlapping communication events.

While differences between the groups were located at certain moments of the clips, there was no evidence of differing brain network activation for most parts of the stimulus data. This may arise from the fact that participants in the ASD group had good cognitive capabilities, which also made it easier for them to participate in the scanning procedure. Nevertheless, many of them struggled in everyday social situations. The young adults with ASD from the same cohort also participated in another study in which their eye movements were tracked while they, in a behavioral test situation, watched a video clip with complex pragmatic content^7^. That study showed that they had some problems in attending their gaze to important social cues, and also some difficulties in understanding the pragmatic content of the video. In addition, in the present study, the video clips were framed in a manner that only the main characters of the scenes were visible, which may have helped the participants better focus their attention. In more demanding settings, resembling everyday social communication situations, the results may have been different. We hypothesize that while pragmatic inferencing is a very complicated process requiring attention to multiple contextual details at the same time, pragmatic challenges of a mild form of ASD do not manifest themselves in simple settings or social contexts, but only when the processing load exceeds a certain threshold^44^. Therefore, the video clips that are used in these kind of studies should be sensitive enough to detect differences between groups. In future studies, there may be possibilities to build more naturalistic test environments utilizing virtual reality (VR) technology.

One of the limitations of this study was that the participants with ASD were diagnosed as children and their diagnosis was not confirmed when they were adults, based on the general thought that ASD is a permanent condition. However, we are aware of the discussion that a small subgroup of individuals who were diagnosed with ASD when they were children, no longer meet the diagnostic criteria in adulthood. In future studies, diagnosis of ASD should be confirmed when the participants are adults. Furthermore, this study involved a rather small number of subjects, and further studies with larger sample sizes are warranted. In future studies, fast fMRI data could be used instead of the BOLD signal for more temporally accurate evaluation of hemodynamic response.

This fMRI-based study including the temporal analysis of brain network components linked to social and pragmatic functioning revealed that processing of complex social situations was for the most part similar between the NT and ASD groups. However, significant differences between the groups were found when the participants were viewing social situations with concurrent verbal and non-verbal communication events. The differences in neural level functioning are presumably related to behavioural markers of ASD concerning comprehension of pragmatically challenging communication situations with multimodal cues.

Pragmatic challenges in ASD may not manifest themselves in simplistic communicative situations, but only when the processing load exceeds a certain threshold due to overlapping contextual cues. Finding moments of atypical social brain network function in ASD, when viewing complex communicative situations, may help to define those particular situations that are extra demanding for individuals with ASD. This is essential when designing effective intervention programs that target alleviating everyday challenges in their lives.

## Methods

### Subjects

This study belongs to a multidisciplinary project at the Oulu University Hospital and the University of Oulu called “Autism spectrum disorders—a follow-up study from childhood to young adulthood” where ASD and control participants from the earlier phases of the study were recruited again during 2014–2015 for clinical and fMRI assessment. The participants of this study were 19 young adults (5 female, 14 male, *M* = 23.6 years, *Md* = 23.0, *SD* = 3.3, range = 19–31) who were diagnosed with ASD as children based on International Classification of Diseases—10th Revision (ICD-10) by WHO^45^ criteria by experienced child psychiatrists or child neurologists utilizing the results of the Autism Diagnostic Interview-Revised (ADI-R)^46^, the Autism Diagnostic Observation Schedule (ADOS)^47^ and other investigations by a multi-professional team at Oulu University Hospital. The control group consisted of 19 young adults who did not have ASD or other neurodevelopmental disorders (4 female, 15 male, *M* = 22.7 years, *Md* = 23.0, *SD* = 2.2, range = 19–29). Both individuals with ASD and their controls were drawn from two earlier studies: a community-based study in the Northern Ostrobothnia Hospital District, Finland initiated in 2000^48,49^ and a clinic-based study at Oulu University Hospital, Finland initiated in 2003^50,51^. Controls were originally recruited in 2006^50^ and re-invited for this study.

Before fMRI scanning, cognitive abilities of the participants were assessed. None of the participants had intellectual disability, and based on the general ability index (GAI) of Wechsler’s intelligence scale^52^, there was no statistically significant difference between the groups (ASD: *M* = 111.3, *Md* = 110.0, *SD* = 13.2; Controls: *M* = 103.7, *Md* = 103.0, *SD* = 13.0; *U* = 232.5*, p* = 0.130) Neither was there statistically significant difference for GAI subscales Verbal Comprehension Index (VCI; ASD: *M* = 111.3, *Md* = 112.0, *SD* = 14.7; Controls: *M* = 104.4, *Md* = 108.0, *SD* = 16.2; *U* = 141.0*, p* = 0.248) or Perceptual Reasoning Index (PRI; ASD: *M* = 108.7, *Md* = 110.0, *SD* = 12.1; Controls: *M* = 102.3, *Md* = 108.0, *SD* = 14.0; *U* = 135.0*, p* = 0.183). The participants completed also the Finnish version of Autism Quotient (AQ) questionnaire^53^, which showed a significant difference between the groups (ASD [*n* = 17, missing 2]: *M* = 19.7, *Md* = 19.0, *SD* = 9.0; Controls [*n* = 16, missing 3]: *M* = 10.6, *Md* = 10.0, *SD* = 4.9; *U* = 217.5, *p* = 0.002) conforming that individuals in the ASD group had more autistic features. The study by Loukusa et al.^54^ has shown that the cut-off score and mean value in AQ in a Finnish sample is much lower than in an English sample (e.g.^53,55^). Based on the above-mentioned study, the mean AQ value in Finnish ASD individuals (*n* = 52) was 22.5 (*SD* = 8.3) and in controls (*n* = 1686) 13.1 (*SD* = 6.4). The suggested cut-off score in Finnish men was 18 and in females 16. It has been suggested that this difference between English and Finnish samples is due to cultural issues.

This study is part of a multidisciplinary research for which a written informed consent was obtained from the participants. The research was approved by the Ethical Committee of Medical Research in the Northern Ostrobothnia District of Finland (79/2012). All procedures performed in the study were in accordance with the 1964 Helsinki declaration and its later amendments or comparable ethical standards.

### Stimulus data

The stimulus of the fMRI study consisted of 7 concatenated video clips (length range 50–84 s) taken from a TV soap opera called *Ruusun aika* from 1990–1991 produced by a Finnish commercial media operator (MTV). The clips from the TV series depict naturalistic social communication situations including members of a certain family, and they were selected for their pragmatic and social content. These particular clips were of interest because they require advanced pragmatic inferencing from the viewer and also complex understanding of verbal and non-verbal communicative cues (see Supplementary Table T1 for contents of the clips).

In the next phase, the video clips were pre-assessed by university students as follows. The idea in the pre-assessment was to expose the pragmatic features that people tend to infer from the video clips. Before the pre-assessment, a team of four researchers of pragmatics formulated a questionnaire where each video clip was described using multiple choices (7 choices). Four of the choices were considered relevant or nearly relevant to the pragmatic content of the clip, two were irrelevant and the last one was an open question. Next, the video clips were shown to 45 students from of the University of Oulu (40 female, 5 male, *M* = 24.1 years, *SD* = 5.4, range = 19–41). After viewing each video clip, the students answered the multiple choice question where they had to select which alternative (A–G) was the best (3 points), the 2nd best (2 points) and the 3rd best match (1 point) for the content of the video. The two choices that gained the most points per clip were selected to best describe the contextual content of that particular clip (see column “Contextual content” in Supplementary Table T1). As an example of the pragmatic content of a video clip, the transcriptions of video clips 2 and 4 can be found in the Supplements (Supplementary Data D1 and D2). Transcriptions of all videos can be requested from the authors of this article.

### Neuroimaging data acquisition and pre-processing of data

The fMRI blood oxygenation level dependent (BOLD) data were obtained using a 32-channel Siemens Skyra 3T scanner and echo-planar imaging pulse sequence (TR = 2150 ms, TE = 28 ms, flip angle = 15°, voxel size = 3 × 3 × 3 mm^3^, matrix size 64 × 64, 45 axial slices for whole-brain coverage). The size of the data was 224 fMRI volumes, which corresponds duration of 7 video clips. In addition, anatomical T1-weighted images (TR = 1900 ms, TE = 2.49 ms, TI = 900 ms, flip angle = 9°, FOV = 240 mm, 0.9 mm cubic voxel) were taken from each participant using a magnetization-prepared rapid gradient-echo (MPRAGE) sequence.

The participants saw the stimulus video(s) on an MRI-compatible screen and heard the sound of the video(s) through a plastic tube in the middle of the ear plug. Soft paddings were installed over the ears for hearing protection and minimizing motion. The volume of a video was adjusted to comfortable hearing level.

A typical FMRIB software library (FSL) pre-processing pipeline^56^ was applied to the BOLD data, including high-pass filtering with a cut-off frequency of 0.008 Hz, motion correction, brain extraction and spatial smoothing (5 mm full width at half maximum Gaussian kernel). The final step included the brain registration to a common Montreal Neurological Institute (MNI) 152 standard space using the anatomical T1-weighted images. There was no statistical difference between the groups in relative (*U* = 203, *p* = 0.525) or absolute (*U* = 192, *p* = 0.751) head motion.

### Analysis of the imaging data: Selecting the independent components for further examination

In the first stage of the analysis, group Independent Component Analysis (gICA), which identifies time-independent brain network components, was performed for the pre-processed 224 volumes of Ruusun aika BOLD data of the control group. The gICA tool (FSL MELODIC) decomposed the fMRI data into 70 independent components revealing the grouping of brain regions that share the same activation pattern in time, which can be described as functional connectivity^57,58^. The gICA maps were used to find individual timecourses and spatial maps of the participants for independent components by using FSL dual regression^59^. Based on visual inspection of gICA components and Harvard-Oxford cortical structural and Juelich histological atlases, three independent components IC4, IC15 and IC27 out of 70 components were chosen for further timecourse inspection (Table 1). The structural overlapping of these independent components is depicted in Fig. 1.

A permutation test with 5000 permutations of each of the selected components revealed that there was no statistically significant difference (*p* < 0.05) between the spatial maps of the ASD and control groups for these independent network components.

### Analysing the BOLD response of the independent components to the stimulus data

In the next stage, the timecourses of the selected ICs were compared between the groups. The statistical analysis of the timecourses showing the BOLD response of the brain network components IC4, IC15 and IC27 was carried out in the MATLAB environment. First, the timecourses of the selected components were normalized for each participant. After normalization, the average value of the timecourses was 0 and all values were between − 1 and 1. The average value of each timepoint within a group (${\stackrel{-}{x}}_{k}$ and ${\stackrel{-}{y}}_{k}$ for the ASD and NT groups, respectively) was calculated for each independent component *k* (*k* = 1, 2, 3). Next, the *p* value of the group difference (${|{\stackrel{-}{x}}_{k}-{\stackrel{-}{y}}_{k}|}^{2}$) for each timepoint was calculated by running a permutation test (10,000 permutations). Fisher’s^60^ combined probability test was run over ICs (*k* = 1, 2, 3) in order to combine the *p* values of the timecourses. After this, Benjamini and Hochberg’s^61^ false discovery rate (FDR 0.05) was used to correct multiple comparisons over timepoints of each video clip.

### Qualitative analysis of events in stimulus data

Finding the interpretation for the brain network activation differences between the groups was based on the temporal relationship between the brain response and the events in the video clip, while taking into account the response latency of the BOLD signal. The interpretation drawn from the events in the stimulus data was based on the qualitative analysis that was done in a team consisting of three researchers in the field of clinical pragmatics (logopedics and psychology).

## Supplementary Information

Supplementary Information.
