# Supplementary material for: Processing of pragmatic communication in ASD: a video-based brain imaging study
Source: Sci Rep. 2020 Dec 10;10:21739. doi: 10.1038/s41598-020-78874-2 (PMC7729953; doi:10.1038/s41598-020-78874-2)
Supplement: Supplementary file 1 — Supplementary Information. [file 41598_2020_78874_MOESM1_ESM.pdf]

Supplements

Title

Processing of pragmatic communication in ASD: a video-based brain imaging study

Authors

Aija Kotila*, Aapo Hyvärinen, Leena Mäkinen, Eeva Leinonen, Tuula Hurtig, Hanna Ebeling, Vesa Korhonen, Vesa J. Kiviniemi, Soile Loukusa

Supplementary Table T1 Contents of the video clips

Video clip	Story content	Contextual content[1]	Inferencing based mainly on following pragmatic features
**1**	Young daughter ("Roosa-Maria") visits a shop to buy chocolate for the birthday cake in order to make her mother ("Marja") happy (at least this is what she insists to the shopkeeper). Her mother is upset when she gets home, because she does not know where she has been.	Marja **being upset** because Roosa-Maria did not act as agreed; **conflict** between Marja and Roosa-Maria	**Verbal:** Marja's indirect verbal expressions of anger<br>**Prosody**: Marja's frustrated and angry tone of voice, high intensity<br>**Body language:** Marja's frustrated and irritated facial expressions (e.g. intensive stare), quick-tempered movements, crossed arms<br>**World and/or social knowledge:** one should keep one's promises
**2**	Grandmother (Senni") and her daughter ("Marja") are having a power struggle over the organization of the kitchen. Marja is not happy that Senni has re-organized the kitchen. Marja's daughter ("Roosa-Maria") watches the situation for a while with her dog, communicates to it using gestures and then leaves with the dog.	Marja's **anger** towards Senni; Senni's **chagrin** because she only tried to help	**Verbal:** Marja directly confronts Senni<br>**Prosody**: Marja's upset voice<br>**Body language:** Marja's angry and Senni's sad facial expressions; Roosa-Maria's silencing gesture to the dog<br>**World and/or social knowledge:** unsolicited organization of the kitchen is not always welcome<br>**Concurrency:** Requires the ability to focus on two parallel communication situations (Marja and Senni; Roosa-Maria and the dog) at the same time and connect information based on them via deduction

**3**	A good-looking young woman (“Ilona”) enters a shop where the young shopkeeper (“Illi”) becomes infatuated with her, not realizing that she is the new employee, because he expected someone with more modest looks.	Illi’s **infatuation** towards Ilona; **confusion** about the identity of Ilona	**Verbal:** Ilona explains her reason for coming; Illi reacts with questions<br>**Prosody**: Illi’s astonished and confused voice<br>**Body language:** Illi’s rigid body language<br>**World and/or social knowledge:** infatuation makes young people act funny
**4**	Son of the family (“Illi”) enters home with his new girlfriend (“Ilona”) wishing to have a private moment together with her, but finding his little sister (“Roosa-Maria") at home. Roosa-Maria talks about a horse facing a mirror, meanwhile the couple decides to leave upstairs. Roosa-Maria follows them upstairs and continues distracting them verbally.	Roosa-Maria **distracting** the young couple; Illi **irritated** because of Roosa-Maria's presence	**Verbal:** Roosa-Maria’s indirect answers to Illi’s direct questions; Roosa-Maria talks about a horse<br>**Prosody**: Illi’s irritated voice<br>**Body language:**<br>Illi’s irritated facial expressions; physical closeness of the young couple; gesturing between the young couple showing suggestion and agreement<br>**World and/or social knowledge:** young couples want privacy<br>**Concurrency:** Requires the ability to focus on two parallel communication situations (Roosa-Maria explaining in front of a mirror; Illi gesturing to Ilona) at the same time and connect information based on them via deduction
**5**	Little sister (“Roosa-Maria”) has taken her big sister’s (“Meri”) belongings without her permission, because Meri is getting married and leaving home. Meri confronts Roosa-Maria about it.	Meri’s **irritation** because of Roosa-Maria's actions; **conflict** between sisters	**Verbal:** Meri directly confronts Roosa-Maria; Roosa-Maria replies defensively<br>**Prosody**: Meri’s angry voice; Roosa-Maria's precocious voice<br>**Body language:** Meri’s irritated facial expressions and quick-tempered movements<br>**World and/or social knowledge:** one should not take other’s belongings without permission

**6**	Mother of the family “Marja” is crying. First, her daughter (“Roosa-Maria”) mentions her horse hobby and makes a comment about Marja’s state and leaves. Next, Marja’s husband (“Esko”) enters and asks about her crying. Marja explains that she is crying because her older daughter (“Meri”) has moved away from home. Esko figures out that Marja has been fighting with Meri, which Marja confirms.	Marja’s **difficulty to let go**; Marja’s **sorrow** because her daughter is leaving home	**Verbal:** Marja explains why she is crying; Esko and Roosa-Maria comment about her crying, suggesting a reason for it<br>**Prosody**: Marja’s sad voice; Esko's calm voice<br>**Body language:** Marja’s sad and crying facial expressions, tears in her eyes<br>**World and/or social knowledge:** letting go is not easy; parting should be done on good terms
**7**	Little sister (“Roosa-Maria”) tells the family about a horse, while her big brother (“Illi”) comments and identifies with the story, accidentally revealing that he is in love with Ilona. Roosa-Maria teases him about it.	Roosa-Maria's and Illi’s direct and indirect expressions of **happiness**, Illi **identifies with** what Roosa-Maria is saying	**Verbal:** Roosa-Maria describes her wonderful experiences with a horse; Illi joins in, indirectly reflecting his own experiences with Ilona<br>**Prosody**: Roosa-Maria's and Illi’s daydreaming voices; Roosa-Maria's teasing voice<br>**Body language:** Roosa-Maria's and Illi’s daydreaming facial expressions<br>**World and/or social knowledge:** people who have recently fallen in love experience strong feelings and think constantly about their loved one

1 based on the assessment by students from the University of Oulu ($n = 45$, $M = 24.1$ years)

Supplementary Data D1 Transcription of the video clip 2

[01:24] **Senni:** what did they say there? ((*inquires in a friendly manner from Marja who pours coffee into Senni's cup*))

[01:25] **Marja:** nothing ((*annoyed*))

[01:26] **Senni:** thank [you] ((*Senni looks downwards while Marja speaks*))

[01:27] **Marja:** [do you think] that you can get a proper answer from the doctors (.) they just beat around the bush

[01:31] **Marja:** where are the cups? ((*starts opening the cupboards*))

[01:32] **Senni:** well, I cleaned the cupboards

[01:33] **Marja**: =what has happened here? ((*opens more cupboards*))

[01:34] **Senni:** they were so dirty so dirty and (.) surely no-one had wiped them (.) since you [wiped them last time]

[01:39] **Marja:** [HOW COME] you have set these kinds of papers here?

[01:41] **Senni:** I set those pape- shelfpapers because they are so pretty when you open the door [we had-]

[01:44] **Marja:** [well no-one] uses shelfpapers anymore they just gather [dust]

[01:48] **Senni:** [look here] I put (.) ((*opens the cupboard to show*)) salt and (.) sugar (.) [and here] you see is flour (.) ((*opens another cupboard*)) just within your reach

[01:52] **Marja:** [did you-]

[01:53] **Marja:** why did you reorganize everything here? ((*At the end of the speech act, the camera shows Roosa-Maria who enters the room holding Nero the dog by the collar. The two women are in the middle of the quarrel and do not notice her.*))

[01:57] **Marja:** THERE IS NO SENSE IN THIS AT ALL ((*Marja's voice can be heard in the background while the camera shows Roosa-Maria who makes a silence gesture to the dog with one finger on her lips*))

[01:59] **Senni:** well I thought that when you are cooking- ((*Senni's voice can be heard but the camera shows Roosa-Maria who whispers something to the dog*))

[02:00] **Marja:** =DON'T THINK (.) this is MY order here ((*Marja's voice can be heard in the background while the camera shows Roosa-Maria, who pulls the dog by the collar to leave the room quietly*))

[02:02] **Marja**: ='cos this is MY kitchen (.) have you forgotten that? ((*the camera now shows Marja and Senni facing each other, Roosa-Maria is no longer visible*))

[02:06] ((*Senni is shown alone, closing the cupboards and looking hurt*))

Supplementary Data D2 Transcription of the video clip 4

[03:22] **Illi:** hi ((*entering home*))

[03:23] **Roosa-Maria:** hi ((*speaking outside the camera view*))

[03:24] **Ilona:** hi ((*she enters after Illi, Roosa-Maria looks at Ilona curiously, Roosa-Maria is wearing a riding helmet*))

[03:27] **Illi:** aha, are you going riding? ((*asking hopefully*))

[03:29] **Roosa-Maria:** it depends ((*Illi gazes at Ilona*))

[03:31] **Illi:** well on what? ((*Illi and Ilona are side by side both looking at Roosa-Maria*))

[03:32] **Roosa-Maria:** on when Ida and daddy are going to pick me up ((*looking at the couple*))

[03:34] **Illi:** Is there anyone else at home?

[03:35] **Roosa-Maria:** it depends

[03:37] **Illi:** come on now Roosa-Maria (.) answer properly ((*requesting facial expression*))

[03:39] **Roosa-Maria:** no (0.7) you know something Illi? (0.9) Sulo has had horse colic several times

((*Roosa-Maria speaks in front of the mirror while she is adjusting her riding helmet, meanwhile Illi nods meaningfully to Ilona and takes her by the hand. Ilona glances at Roosa-Maria to make sure she does not notice what is going on between the couple. The couple sneaks secretly upstairs behind Roosa-Maria's back.*))

[03:45] **Roosa-Maria:** do you know Illi what colic is? ((*she turns to the couple to notice that they have left*))

[03:48] **Roosa-Maria:** hmph (0.9) Illi:: (0.7) come on Nero ((*she calls the dog and walks upstairs entering Illi's room where the couple sits on the bed*))

[04:01] **Illi:** aach ((*annoyed grunt, stops making out with Ilona*))

[04:02] **Roosa-Maria:** just like that it said

[04:03] **Illi:** huh (.) who? ((*frustrated*))

[04:05] **Roosa-Maria:** that Sulo

[04:06] **Illi:** argh ((*more annoyed grunt*))

[04:07] **Roosa-Maria:** have you been sniffing?

[04:08] **Illi:** @what@ ((*frustrated and angry*))

[04:09] **Roosa-Maria:** because you grunt like that (.) Sulo always gets colic when he sniffs ((*clearly not understanding what colic or sniffing means))*

[04:13] ((*Illi and Ilona gaze at each other*))

[04:14] **Ilona:** I don't understand a thing now ((*looking confused*))

[04:15] **Roosa-Maria**: because you are from the countryside ((*looking precocious*))

[04:17] **Illi:** hhh (0.7) Roosa-Maria dear why won't you go riding now ((*speaks gently*))

[04:21] **Roosa-Maria:** ok ((*speaking outside the camera view, footstep sounds when leaving*))

[04:24] ((*Illi lifts his finger to his lips making a "silence" gesture to Ilona. The couple sneaks behind Roosa-Maria's back into another room. Roosa-Maria starts to descend the stairs*))

[04:31] **Roosa-Maria:** oh yeah (.) I forgot to explain what sniffing means ((*turns around in the staircase in order to return to Illi's room*))
